# Supplementary material for: Effects of non-aversive versus tail-lift handling on breeding productivity in a C57BL/6J mouse colony
Source: PLoS One. 2022 Jan 28;17(1):e0263192. doi: 10.1371/journal.pone.0263192 (PMC8797240; doi:10.1371/journal.pone.0263192)
Supplement: S2 File — (DOCX) [file pone.0263192.s002.docx]

**SUPPLEMENTARY FILE 2**

**S2.1 Text: University of Florida C57BL/6J Breeding Colony**

The C57BL/6J breeding colony was established in 2005, and is maintained internally via Animal Care Services. The mice were obtained from The Jackson Laboratory, stock #000664. The colony consists on average of 100 breeding pairs maintained in a continuous monogamous breeding scheme, with an average 7-month turnover, for approximately 200 pairs per year. Genetics are refreshed from the Jackson Laboratory every five generations. Colony production averaged 3,935 mice per year for the previous three years (2018-2020), with an estimated 2018 productivity index of 1.14.

**S2.2 Text: Rodent Health Surveillance Testing**

Dirty bedding sentinel testing was performed quarterly from July 1^st^, 2020 through December 31^st^, 2020. Briefly, dirty bedding of approximately 1 teaspoon in volume from every dirty cage on a rack was transferred into a designated sentinel cage during each routine cage change. Sentinel cages were located at the lowest row and farthest right position on each rack. Each cage was stocked with two female CD-1 IGS mice. Mice were bled quarterly for serum testing via facial vein puncture; animals were rotated between bleeding events. Serum samples were submitted for analysis to Charles River Laboratories Diagnostics. Sentinels were euthanized following 3 quarters of testing.

Environmental PCR testing was performed quarterly from July 1^st^, 2020 through March 31^st^, 2021. Swabs were taken from ventilated rack exhaust plenums. Two weeks prior to a scheduled rack change, when a given rack had been in service for at least 6 weeks, racks were disconnected from the air supply, and the exhaust plenum opened. Adhesive swabs were rolled on the inner surface of the horizontal exhaust plenum ports, samples aggregated by rack, and submitted by room. Swab samples were submitted for analysis via Charles River Laboratories Diagnostics.

| **University of Florida Breeding Core Mouse Pathogen Exclusion List** | | |
| --- | --- | --- |
| **Viruses** | **Bacteria & Fungi** | **Parasites & Protozoa** |
| - Murine Norovirus - Ectromelia (ECTRO) - Epizootic Diarrhea of Infant Mice Virus (EDIM) (Rotavirus) - Lymphocytic choriomeningitis virus (LCMV) - Minute Virus of Mice (MVM) (MMV) - Mouse Adenovirus 1,2 (MAV 1 & 2, Mad) - Mouse cytomegalovirus (MCMV) - Mouse Hepatitis Virus (MHV) - Mouse Parvovirus (MPV) (NS1) - Pneumonia Virus of Mice (PVM) - Reovirus 3 (REO) - Sendai virus (Send) - Theiller’s Murine Encephalomyelitis Virus (GDVII) (TMEV) | - *Helicobacter* sp - CAR bacillus (CARB) - *Citrobacter rodentium* (CITRO) - *Clostridium piliforme* (CPIL) - *Corynebacterium kutscheri* (CKUT) - *Mycoplasma pulmonis* (MPUL) - *Salmonella* sp. (SALM) - *Streptobacillus moniliformis* (SMONO) | - Fur mites (Ecto for Ectoparasites) - Pinworms (Asp for Aspicularis, Syph for Syphacia) |

**S2.3 Text: Statistical analysis**

Power (1 - β) was estimated from the non-centrality parameter of the non-central F-distribution (given the Poisson distribution for litter size, and α = 0.05) obtained from SAS *proc glimmix* and with functions FINV and PROBCHI [1, 2]. The power for an operational difference of one extra pup per pair for an expected baseline production of 30 pups and a sample size of 30 pairs per arm was 0.11. To achieve power > 0.8 for formal significance testing with a fixed sample size of 30 pairs per arm, the study would have required a much larger-than-anticipated effect size of 4-5 extra pups weaned per pair (or one extra pup per litter). Alternatively, to detect a difference of one pup per pair, the study would require at least 105 breeding pairs per arm, or >210 pairs.

**S2.4 Text: SAS code**

data mice;

* trt = treatment identifiers, mean = expected mean litter size (counts) for each arm;

input trt $ mean;

n=65; *change to desired sample size per treatment arm;

do obs=1 to N;

output;

end;

datalines;

*change to expected mean litter size (counts) for each arm;

control 6

test 7

;

run;

proc print data=mice;

run;

proc glimmix data= mice;

class trt;

model mean = trt / chisq link=log dist=poisson;

contrast 'control vs experimental' trt 1 -1 / chisq ;

ods output tests3=F_overall contrasts=F_contrasts;

run;

data power;

set F_overall F_contrasts;

nc_parm=numdf*Fvalue; *calculate non-centrality parameter;

alpha=0.05;

F_Crit=Cinv(1-alpha,numdf,0);

Power=1-probchi(F_crit,numdf,nc_parm);

proc print data=power;

run;

**References**

1. Stroup, W.W. Living with Generalized Linear Mixed Models. SAS Global Forum 2011.

2. Littell, R.C., Milliken, G.A., Stroup, W.W., Wolfinger, R.D., Schabenberger, O. SAS for Mixed Models. 2nd ed. Cary, North Carolina, United State of America: SAS Institute; 2007. p. 479-97.

**S2.5 Text: Supplementary results**

S2.5 Table 1: Count data and distribution of litter sizes (pups born, weaned) by parity

|  | | Tunnel-handled | | Tail-lift with forceps | | |
| --- | --- | --- | --- | --- | --- | --- |
| **Number of breeding pairs** | | 29 | | 30 | | |
| **Total number of litters produced** | | 141 | | 145 | | |
| **Number of entire litters lost^1^** | | 24 | | 34 | | |
|  | | *Pups born* | *Pups weaned* | *Pups born* | | *Pups weaned* |
| **Total** | | 1006 | 792 | 944 | | 726 |
| **Litter Parity** | |  | |  | | |
| 1 | | 241 | 210 | 224 | | 197 |
| 2 | | 202 | 165 | 208 | | 169 |
| 3 | | 175 | 124 | 180 | | 133 |
| 4 | | 163 | 133 | 157 | | 94 |
| 5 | | 124 | 86 | 94 | | 75 |
| 6 | | 84 | 66 | 64 | | 55 |
| 7 | | 17 | 8 | 17 | | 3 |
| ^1^ Includes litters for 3 dams found dead or euthanized for dystocia | | | |  |  |  |

S2.5 Table 2: Generalized hierarchical model results for pups born and weaned per litter for 286 litters from 59 breeding pairs of C57BL/6J mice.

| **Variable** | **Level** | **Coefficient** | **SE** | **p** |
| --- | --- | --- | --- | --- |
| *Pups born* |  |  |  |  |
| **Intercept** |  | 1.807 | 0.035 | <0.0001 |
| **Intervention** | Tail-lift with forceps | 0 |  |  |
|  | Tunnel handling | 0.038 | 0.046 | 0.41 |
| **Variance** | Tail-lift with forceps | 1.033 | 0.117 | <0.0001 |
|  | Tunnel handling | 0.833 | 0.094 | <0.0001 |
| **ρ^1^** | Tail-lift with forceps | 0.079 | 0.089 | 0.89 |
|  | Tunnel handling | 0.012 | 0.084 | 0.37 |
| *Pups weaned* |  |  |  |  |
| **Intercept** |  | 1.551 | 0.049 | <0.0001 |
| **Intervention** | Tail-lift with forceps | 0 |  |  |
|  | Tunnel handling | 0.064 | 0.065 | 0.33 |
| **Variance** | Tail-lift with forceps | 1.823 | 0.222 | <0.0001 |
|  | Tunnel handling | 1.488 | 0.183 | <0.0001 |
| **ρ^1^** | Tail-lift with forceps | -0.208 | 0.088 | 0.02 |
|  | Tunnel handling | -0.205 | 0.088 | 0.02 |

^1^ Indicates the correlation in number of pups born and weaned with parity
