# Supplementary material for: Effects of non-aversive versus tail-lift handling on breeding productivity in a C57BL/6J mouse colony
Source: PLoS One. 2022 Jan 28;17(1):e0263192. doi: 10.1371/journal.pone.0263192 (PMC8797240; doi:10.1371/journal.pone.0263192)
Supplement: Attachment — Submitted filename: Effects of NAH on B6 Breeding colony_Response_2.docx [file pone.0263192.s004.docx]

Additional Editor Comments:

1. Please include line references in the revised version, and refer to these in your response letter.

2. This paper applies different terms for the handling techniques than most papers in the field. Handling mice using a tunnel is usually referred to as tunnel handling, so please use that term here. Standard handling in most studies of handling method is referred to as tail handling / tail picking. However, as far as I know I have never seen tail handling being reported to be done using forceps, so your method seems to be a little different here. Perhaps it can be referred to as forceps tail picking.

**Author response**: Acronyms for treatment groups were removed in full throughout the manuscript. Non-aversive handling was changed to “tunnel handling” or “tunnel-handled” where appropriate. Figures 1 & 2 were also adjusted to have consistent labelling. Tail-lift with forceps is a very common (that is nearly ubiquitous) method of cage change handling in the United States and much of North America. This is discussed in more depth in (Doerning 2019), and is now referenced in the manuscript.

3. Page 3 last sentence (the one that continues on page 4): please add a reference for this statement.

**Author response**: Citation added, (Orso, 2017), page 4 line 92.

4. Page 6, last paragraph: Can you separate litters disappearing from litters having to be euthanized and provide separate figures? Please provide more information on the situations of euthanasia - what was the reason to decide to euthanize a litter? Also, were the three litters from dams found dead or euthanized because of dystocia included in the analysis?

**Author response:** Table 1 (page 5 line 124-125) was expanded to include pups euthanized and litters lost. No entire litter was euthanized, only individual pups meeting stated endpoint criteria on page 14 lines 423-432 were euthanized. Added additional clarifying information for exact pup endpoint criteria (page 14 lines 427-429). Litters from dams that were found dead or euthanized for health concerns were included for analysis, as the analysis was conducted as ‘intention to treat’. This was added to page 14 lines 431-432 and page 15 lines 449-450.

5. Page 7 first paragraph: Please add a definition for recurrent litter loss.

**Author response:** Added, page 6 line 168 and page 15 line 449-450.

6. Discussion, first paragraph: Does the definition "operationally significant (albeit statistically non-significant) increases" apply generally to the results? You address this further on in the discussion (bottom of page 8), but it becomes a little confusing as you seem to apply more than one definition of operationally significant. See "statistically-significant difference of one extra pup per litter, rather than one extra pup per pair, which was the operational criterion" versus "one extra pup weaned per pair and the magnitude of reduction in litter loss were predetermined to be operationally significant differences". Please see if you can express this in a more straightforward way.

**Author response**: Reworded page 2 lines 31-33, page 6 lines 133-138.

7. Page 8 line 3: There really is very limited support in literature for infanticide being the cause of litter loss. Our most recent paper adds to the evidence that infanticide rarely is the cause when pups die: *Animals* **2021**, *11*(8), 2327; [https://doi.org/10.3390/ani11082327](https://urldefense.proofpoint.com/v2/url?u=https-3A__doi.org_10.3390_ani11082327&d=DwMGaQ&c=sJ6xIWYx-zLMB3EPkvcnVg&r=3JAg_Zf6Q3v7FKYLWyaB6A&m=EcJqBFHbTZqO-3tvRqAvhqasjZPmSL4SC-rhYbtj8PoDHwLnZezznSJa9ncu7Wkv&s=pnl1mxkCRqk6X7MoU_Ozlg5owokQEbLSyWOz4XC6bM4&e=)

**Author response**: We agree and have removed this phrase.

8. Page 8 middle of the page: The lack of an association between pup mortality and primiparity was also found by us in a retrospective study of breeding data Reproduction in Domestic Animals. doi: 10.1111/j.1439-0531.2012.02147.x. [https://onlinelibrary.wiley.com/doi/epdf/10.1111/j.1439-0531.2012.02147.x](https://urldefense.proofpoint.com/v2/url?u=https-3A__onlinelibrary.wiley.com_doi_epdf_10.1111_j.1439-2D0531.2012.02147.x&d=DwQGaQ&c=sJ6xIWYx-zLMB3EPkvcnVg&r=3JAg_Zf6Q3v7FKYLWyaB6A&m=EcJqBFHbTZqO-3tvRqAvhqasjZPmSL4SC-rhYbtj8PoDHwLnZezznSJa9ncu7Wkv&s=pPgbK6BuaeYiaCR3bj3aD-8QZOVvicZnohIMCnXurZ4&e=)

**Author response:** This citation was added. It was in a previous draft but was inadvertently omitted during revision. Thank you for the reminder.

9. The standard handling method in your study is tail handling using forceps. I haven't heard of forceps handling in a very long time, and I wonder how generalisable your results are to situations where standard handling is tail handling with the human hand. Please discuss this.

**Author response:** As stated previously cage change utilizing forceps is very common in the United States, and is discussed more in depth in (Doerning, 2019). We expanded upon this topic on page 8 lines 273-280. This paper intended to compare our previous institutional standard method to tunnels, with the goal of institutional change of practice.

[Note: HTML markup is below. Please do not edit.]

Reviewers' comments:

Reviewer's Responses to Questions

**Comments to the Author**

1. Is the manuscript technically sound, and do the data support the conclusions?

The manuscript must describe a technically sound piece of scientific research with data that supports the conclusions. Experiments must have been conducted rigorously, with appropriate controls, replication, and sample sizes. The conclusions must be drawn appropriately based on the data presented.

Reviewer #1: Yes

2. Has the statistical analysis been performed appropriately and rigorously?

Reviewer #1: Yes

3. Have the authors made all data underlying the findings in their manuscript fully available?

The [PLOS Data policy](https://urldefense.proofpoint.com/v2/url?u=http-3A__www.plosone.org_static_policies.action-23sharing&d=DwMGaQ&c=sJ6xIWYx-zLMB3EPkvcnVg&r=3JAg_Zf6Q3v7FKYLWyaB6A&m=EcJqBFHbTZqO-3tvRqAvhqasjZPmSL4SC-rhYbtj8PoDHwLnZezznSJa9ncu7Wkv&s=kyT1GQaW9lh8Gzqv8pyryzIBX5LKAXIwnCDTykGHHOQ&e=) requires authors to make all data underlying the findings described in their manuscript fully available without restriction, with rare exception (please refer to the Data Availability Statement in the manuscript PDF file). The data should be provided as part of the manuscript or its supporting information, or deposited to a public repository. For example, in addition to summary statistics, the data points behind means, medians and variance measures should be available. If there are restrictions on publicly sharing data—e.g. participant privacy or use of data from a third party—those must be specified.

Reviewer #1: Yes

4. Is the manuscript presented in an intelligible fashion and written in standard English?

PLOS ONE does not copyedit accepted manuscripts, so the language in submitted articles must be clear, correct, and unambiguous. Any typographical or grammatical errors should be corrected at revision, so please note any specific errors here.

Reviewer #1: Yes

5. Review Comments to the Author

Please use the space provided to explain your answers to the questions above. You may also include additional comments for the author, including concerns about dual publication, research ethics, or publication ethics. (Please upload your review as an attachment if it exceeds 20,000 characters)

Reviewer #1: Overall comments
I would like to thank the authors for a well written manuscript. Their findings are interesting and novel and have the potential to have significant implications for mouse welfare and economics of mouse breeding colonies. Although their findings remain statistically insignificant, the authors include this caveat accordingly, but present convincing arguments with regards to the economic benefits regardless of the small effect size.

Minor comments/suggestions
The manuscript would benefit from a clearer representation of the data in the forms of its table and figures. Although I appreciate the information portrayed in Table 2, I don’t think this adds to the results section.

**Author response**: Table 2 has been moved to supplemental materials, S2.5 Table 1.

Furthermore, the findings of one extra pup born and weaned with NAH as outlined in Figure 1 is not immediately clear. Although I appreciate its useful to demonstrate the spread of the data in terms of litter size and occurrence frequency, perhaps the addition of a cumulative number of pups for both would present the findings in a clearer manner.

**Author response**: We agree, and have revised figure 1 to show cumulative totals for pups born and weaned per pair.

The same applies to the other percentage data presented in the main text, perhaps a summary table of the main findings would aid the reader.

**Author response:** We agree, and have revised table 1 to address reviewer comments, and moved Table 2 and count data to supplemental results, S2.5 Table 1 & 2.

I would appreciate if the authors could include some discussion as to their ‘choice’ or ‘requirement’ for why tail handling is facilitated by forceps as this is not the standard tail handling comparison utilized in most (if not all) the current literature focused on NAH. They could then speculate whether tail handling using forceps is likely to have a greater impact on their measured effects compared to the use of fore finger and thumb.

**Author response:** As stated for the previous reviewer, tail-lift with forceps is an extremely common method in the US, and is often the institutional standard due to concern over biosecurity, prevention of cross-contamination, and operator injury (albeit there is evidence showing that this method increases the incidence of operator repetitive injury!). Although we have little hard information as to the actual prevalence of tail handling with forceps in US academic institutions, we have plenty of anecdotal evidence to suggest it is unfortunately a widespread practice in the US. We have provided a reference in support (Doerning, 2019, page 8 lines 273 – 280).

The authors should also be careful in their phrasing with regards to their data showing direct benefits to animal welfare as they do not have any direct measures of welfare presented in their manuscript (pp 7 discussion line 4). I appreciate that environment (and handling) are important factors of stress which has been shown to influence dam welfare, the fact that there is no data confirming greater stress levels means careful wording is needed.

**Author response:** Changed “welfare benefits” to “productivity” and added a sentence (page 9, line 232-233) to suggest that welfare benefits associated with the reduction in possibly preventable pup deaths.

Methods section – there should be the inclusion of information with regards to the handling of adult mice taken forward for breeding. Were these tail handled up until the point of breeding?

**Author response**: Yes, as it was the institutional method of mouse handling during routine husbandry care at the time, so this is an unavoidable confound. Added a sentence explaining this, (page 13, line 398-399).

Tuesday, January 11, 2022

The manuscript has been thoroughly revised and all comments in the previous round of reviews have been addressed, and there is only one minor issue that need clarifying:

On lines 163-164 and on lines 175-176, you write about an increase in the proportion of all litters successfully weaned and a decrease in likelihood that an entire litter was lost prior to weaning. Can you please clarify how these results are independent? I may be misunderstanding this, but it seems to me that either a litter is successfully weaned or it is lost prior to weaning, and therefore these two results are dependent on each other, which would make it redundant to report both.

**Author response**: We agree. We have altered the language on these sentences to be more clear when discussing all litters produced by a single dam and all litters for an entire treatment group.
